# Perception of the ethical climate among healthcare professionals in an emergency room from southern Brazil

**DOI:** 10.15649/cuidarte.4196

**Published:** 2024-12-19

**Authors:** Taís Carpes Lanes, de Lima Dalmolin Graziele, Augusto Maciel da Silva, Camila Antunez Villagran, Carolina da Silva Caram, Tânia Solange Bosi de Souza Magnago

**Affiliations:** 1 Universidade Federal de Santa Maria, Santa Maria, RS, Brasil. taislanes_rock@hotmail.com Universidade Federal de Santa Maria Universidade Federal de Santa Maria Santa Maria, RS Brasil taislanes_rock@hotmail.com; 2 Universidade Federal de Santa Maria, Santa Maria, RS, Brasil. graziele.dalmolin@ufsm.br Universidade Federal de Santa Maria Universidade Federal de Santa Maria Santa Maria, RS Brasil graziele.dalmolin@ufsm.br; 3 Universidade Federal de Santa Maria, Santa Maria, RS, Brasil. augustolavras@gmail.com Universidade Federal de Santa Maria Universidade Federal de Santa Maria Santa Maria, RS Brasil augustolavras@gmail.com; 4 Universidade do Rio Verde, Rio Verde, GO, Brasil. camilaantunezvillagran@gmail.com Universidade do Rio Verde Universidade do Rio Verde Rio Verde, GO Brasil camilaantunezvillagran@gmail.com; 5 Universidade Federal de Minas Gerais, Belo Horizonte, MG, Brasil. caram.carol@gmail.com Universidade Federal de Minas Gerais Universidade Federal de Minas Gerais Belo Horizonte, MG Brasil caram.carol@gmail.com; Universidade Federal de Santa Maria, Santa Maria, RS, Brasil. tania.magnago@ufsm.br Universidade Federal de Santa Maria Universidade Federal de Santa Maria Santa Maria, RS Brasil tania.magnago@ufsm.br

**Keywords:** Ethics, Professional, Organizational Culture, Nursing, Hospital, Physicians, Emergency Medical Services, Ética Profesional, Cultura Organizacional, Enfermería, Médicos, Hospital, Servicios Médicos de Urgencia, Ética Profissional, Clima Organizacional, Enfermagem, Médicos, Hospital, Serviços Médicos de Emergência

## Abstract

**Introduction:**

The ethical climate is defined as the shared perception among healthcare professionals of what is ethically correct behavior and how to deal with ethical issues.

**Objective:**

To evaluate the perception of the ethical climate among health professionals working in an emergency room.

**Materials and Methods:**

Cross-sectional study carried out with healthcare professionals working in the emergency room of a university hospital in southern Brazil. Collection took place in January 2024 through the Positive Ethical Climate Promotion Platform, through the Hospital Ethical Climate Survey-Brazilian Version instrument. The data was organized and analyzed on the Positive Ethical Climate Promotion Platform, applying descriptive statistics.

**Results:**

71 health professionals participated in the research, in which the general ethical climate was classified as positive (M=3.73; SD=0.60). The factors were evaluated as positive, except for the medical factor (M=3.23; SD=1.16), classified as negative. Descriptively, female professionals (p=0.063), with a partner (p=0.508), aged over 42 years (p=0.047) and with training time over 17 years (p=0.072) presented high averages for positive ethical climate, however, only age showed a significant association.

**Discussion:**

Despite the positive assessment of the general ethical climate, the importance of self-reflection and self-awareness when making ethical decisions in care is understood.

**Conclusion:**

The general ethical climate and its factors were evaluated as positive, however, the medical factor was perceived as negative.

## Resumen

**Introducción:**

El clima ético se define como la percepción compartida entre los profesionales de la salud sobre lo que es un comportamiento éticamente correcto y cómo abordar las cuestiones éticas.

**Objetivo:**

Evaluar la percepción del clima ético entre los profesionales de la salud que trabajan en una sala de emergencia.

**Materiales y métodos:**

Estudio transversal realizado con profesionales de la salud que actúan en la sala de emergencia de un hospital universitario del sur de Brasil. La recolección se realizó en enero de 2024 a través de la Plataforma de Promoción del Clima Ético Positivo, a través del instrumento Encuesta de Clima Ético Hospitalario-Versión Brasileña. Los datos fueron organizados y analizados en la Plataforma de Promoción del Clima Ético Positivo, aplicando estadística descriptiva.

**Resultados:**

Participaron de la investigación 71 profesionales de la salud, en la que el clima ético general fue clasificado como positivo (M=3,73; DE=0,60). Los factores fueron evaluados como positivos, excepto el factor médico (M=3,23; DE=1,16), clasificado como negativo. Descriptivamente, profesionales del sexo femenino (p=0,063), con pareja (p=0,508), con edad mayor a 42 años (p=0,047) y con tiempo de formación superior a 17 años (p=0,072) presentaron promedios altos de clima ético positivo, sin embargo, sólo la edad mostró una asociación significativa.

**Discusión:**

A pesar de la valoración positiva del clima ético general, se comprende la importancia de la autorreflexión y la autoconciencia a la hora de tomar decisiones éticas en el cuidado.

**Conclusiones:**

El clima ético general y sus factores fueron evaluados como positivos, sin embargo, el factor médico fue percibido como negativo.

## Resumo

**Introdução:**

A pandemia de COVID-19 levou a uma alta prevalência de ansiedade e depressão entre os profissionais de saúde.

**Objetivo:**

Avaliar a percepção do clima ético entre os profissionais de saúde atuantes em um pronto-socorro.

**Materiais e métodos:**

Estudo transversal realizado com profissionais de saúde atuantes em pronto-socorro de um hospital universitário do sul do Brasil. A coleta ocorreu em janeiro de 2024 através da Plataforma de promoção do clima ético positivo, por meio do instrumento Hospital Ethical Climate Survey-Versão Brasileira. Os dados foram organizados e analisados na Plataforma de promoção do clima ético positivo, aplicando estatística descritiva.

**Resultados:**

Participaram da pesquisa 71 profissionais de saúde, em que o clima ético geral foi classificado como positivo (M=3,73; DP=0,60). Os fatores foram avaliados como positivos, exceto, o fator médico (M=3,23; DP=1,16), classificado como negativo. De forma descritiva, profissionais do sexo feminino (p=0,063), com companheiro (p=0,508), idade acima de 42 anos (p=0,047) e com tempo de formação acima de 17 anos (p=0,072) apresentaram médias elevadas para o clima ético positivo, no entanto, somente a idade apresentou associação significativa.

**Discussão:**

Apesar da avaliação positiva do clima ético geral, compreende-se a importância de uma autorreflexão e autoconsciência mediante a tomada de decisão ética na assistência.

**Conclusões:**

O clima ético geral e os seus fatores foram avaliados como positivo, no entanto, o fator médicos foi percebido como negativo.

## Introduction

Ethical climate is defined as the shared perception among healthcare professionals of what constitutes ethically correct behavior and how to deal with ethical issues. In this context, management provides the basis and direction for ethical behavior among employees within their organization^1^,^2^. 

This phenomenon can be classified as positive or negative. A positive perception occurs when health professionals are able to act in care based on guidelines and codes of ethics of their category and institution, providing support for the moral deliberation of their managers^3^. Shared decisions and a good communication structure enable a positive perception of the ethical climate, since this attitude seeks the well-being of the patient based on professional accountability, including the code of ethics and "ethical team time out". The “ethical team time out” refers to the time for participatory decision-making between the team and the patient, strengthening the patient’s autonomy^4^. 

Studies have reported the prevalence of a positive perception of the ethical climate among health professionals from different sectors, including pediatric units/obstetric center (3.82 ± 0.63)^5^, hemodialysis unit (3.63 ± 1.03)^6^ and inpatient clinical units (3.70 ± -0.62)^7^, with the exception of critical care, especially the emergency room (3.4 ± -0.68)^7^. Critical units refer to the context of intensive care and emergency room work with care for highly complex patients, in which decisions with enormous medical and ethical implications are required every day^4^ . 

Despite the excessive workload of employees, patients must be cared for in all their dimensions and completeness. Care based on standardized procedures according to ethical guidelines is useful in decision-making, as long as it is trained and exemplified in an interdisciplinary and interprofessional manner^8^. 

According to the literature, critical units, especially emergency rooms, are characterized by a negative ethical climate, which indicates difficulties in interpersonal relationships, especially between doctors and nurses, as well as little or no ethical support in decision-making^2^. The team is recommended to take some time to evaluate the therapeutic decision, during which the patient's wishes and the health team's recommendations are reviewed in a structured manner, promoting ethical and quality care^4^. A negative ethical climate can affect care, since it is associated with the intention to abandon the profession (β=−0.053; 95% CI (−0.083 to –0.029); p<0.001) and with the quality of care (β=0.049; )95% CI (0.028; 0.077); p <0.001^9^. 

Related to achieving an ethical climate in teamwork, a work environment with fewer ethical conflicts and greater professional engagement can lead to better quality of patient care and greater organizational dedication. Given the serious tensions and ethical dilemmas that continually arise in the emergency room context, it is important to promote a positive ethical climate, which has an impact on job satisfaction and work-related risks^10^. 

Despite the difficulties experienced by healthcare teams when faced with ethical issues and dilemmas, this is still a partially unexplored field of research, as few studies have designed intervention programs to promote a positive ethical climate. The interventions found in the literature are associated with the inclusion of codes of ethics, reflection and ethical education through lectures and short courses^8^,^11^. However, no interventions were found to promote a positive ethical climate^12^. 

In contrast, the Positive Ethical Climate Promotion Platform (PEP) developed and validated in Brazil seeks to promote a positive ethical climate in the emergency room. It is a management tool with different functions, especially for diagnosing the ethical environment and suggesting strategies for promoting an ethical climate, which was used in this study. 

Furthermore, it selects strategies to promote a positive ethical climate, according to the factors that make up the HECS-VB, namely: peers, doctors, patients, managers and hospitals. Thus, the objective was to evaluate the perception of the ethical climate among health professionals working in an emergency room. 

## Materials and Methods

This is a cross-sectional study, carried out in a university hospital in southern Brazil with emergency room health professionals. 

The PEP is a computerized platform that was applied in the emergency room of a public teaching hospital located in the central region of Rio Grande do Sul, Brazil. The PEP was developed in 2024 and obtained good reliability and technical quality for its application in emergency room^13^. The platform allows the computerization of the HECS-VB instrument^14^, sociodemographic and labor data, as well as selecting strategies to improve the ethical climate based on the results by factors. In this study, only the PEP was used to diagnose the ethical climate. The PEP comes from a doctoral thesis entitled “Platform for promoting a positive ethical climate in emergency rooms” which sought its development and evaluation of technical quality. 

The ethical climate is assessed using the five factors of the HECS-VB^15^ , which are: peers (professionals in the same category), physicians, patients, managers (immediate management) and hospital (management at the hospital level)^1^,^15^. All healthcare professionals in the emergency room were invited to complete the research instruments. The inclusion criterion was to have been working in the institution and unit for at least one month. Professionals who were absent for any reason during the data collection were excluded. 

Currently, it is estimated that there are approximately 121 health professionals (doctors, nurses and nursing technicians) working in the emergency room. It was decided to invite all health professionals to participate in the research, but the viable population was 100, since 21 of them were on vacation or sick leave. 

Before starting the collection, the objectives of the research, its benefits and risks, and the collection period were explained to the participants, to keep them informed about this stage. Data collection took place in January 2024, after the project was approved by the institution's Research Ethics Committee (REC). Health professionals accessed the sociodemographic and labor instruments and the HECS-VB via access to the PEP on computers, tablets, or smartphones. 

Two members of the research group were trained by the author and advisor to assist in the application of the platform. For training, a manual was prepared discussing how to approach participants and general explanations about the instrument and system. The collection took place in a private room in the emergency room, where the Free and Informed Consent Form (FICF) was accessed via the platform, guaranteeing the right to privacy and the right to withdraw from the research at any time, or to permanently cease participation, according to the participant's wishes, with no public exposure of any person or their information, in addition to presenting the confidentiality agreement. 

Two instruments were used, the first consisting of sociodemographic data (age, sex, marital status and having children) and labor data (professional category, education, employment relationship, emergency department sectors and time since professional training). The second, the HECS-VB instrument to assess the ethical climate, which was developed by Linda Olson in 1998^1^, in Chicago, United States. It was validated for Brazil by Lanes^15^ in 2023 to assess the perception of health professionals about the ethical climate in the workplace. 

This instrument consists of 26 items organized into five factors: peers (colleagues: 4 items); patients (4 items); managers (immediate management: 6 items); hospital (institution management: 6 items) and physicians (6 items). The Likert scale consists of five-point response options: 1 = almost never true; 2 = rarely true; 3 = sometimes true; 4 = often true and 5 = almost always true^1^,^15^. The response time for both instruments was on average 10 to 15 minutes. The cut-off point for assessing the ethical climate is: <3.5 = negative ethical climate and ≥3.5 = positive ethical climate^15^. 

The reliability of the internal consistency of the validated instrument was measured by Cronbach 's alpha coefficient, reaching a value of 0,93 for the overall HECS-VB and varying among the five factors: peers (0.72); patients (0.59). managers (0.91); hospital (0.80) and physicians (0.81)^15^,^16^. Thus, the HECS-VB proved to be valid and reliable, with its reliability higher than 0,70^17^ . 

The data were organized and analyzed via PEP, through a React JS interface with Cloud Firestore, which is a cloud-based NoSQL database server that stores and synchronizes data. To measure the ethical climate, sociodemographic and labor data, descriptive statistics were used, with measures of position (mean, median) and dispersion (standard deviation, interquartile range) according to the normality distribution of the data (Kolmogorov -Smirnov test). Student 's t-test was performed on sociodemographic/labor variables and general ethical climate for dichotomous variables and ANOVA for polytomous variables^17^. The data collected in full are available for free access and consultation on Mendeley Data^18^. 

The study was approved by the REC according to opinion number: 5,463,055 on June 10, 2022. The rules and guidelines that regulate research with human beings established through Resolution No. 466/12 were followed, as well as Resolution 510/2016 that regulates the registration of consent in the digital environment^19^,^20^. The professionals who agreed to participate signed the Free and Informed Consent Form, in two copies, with one copy for each one, guaranteeing privacy and the right to withdraw from participation in the research at any time. 

## Results

A total of 71.0 (71) health professionals participated in the research, of which 32.39 (23) were nurses, 46.47 (33) were nursing technicians, 4.22 (3) were doctors and 16.90 (12) were medical residents, working in the adult emergency room 80.28 (57) and pediatric emergency room 19.71 (14). 

Table 1 shows the descriptive statistical results (mean and standard deviation) of the HECS-VB instrument. 

**Table 1:** Descriptive statistics of Ethical Hospital Climate Survey – Brazilian version. Santa Maria, RS, 2024, (n=71)

HECS-VB factors and items	A (DP)
General ethical climate	3.73(0.60)
Pairs	4.20(0.84)
1. My colleagues pay attention to my concerns about patient care.	4.08(0.87)
10. My colleagues help me with difficult issues/problems in patient care.	4.24(0.80)
18. I work with competent colleagues.	4.21(0.83)
23. Safe patient care is provided on my unit.	4.35(0.76)
Patients	3.86(0.88)
2. Patients know what to expect from the care provided to them.	3.56(0.95)
6. Nurses have access to the information needed to resolve an is-sue/problem in patient care.	3.90(0.85)
11.Nurses use the information needed to solve a specific issue/problem in patient care.	4.07(0.83)
19. Patients' wishes are respected.	3.93(0.72)
Managers	3.92(1.09)
3. When I am unable to decide what is right or wrong in a patient care situation, my boss helps me.	3.87(1.11)
7. My boss supports me in my decisions about patient care.	3.93(0.96)
12. My boss listens to what I say about issues/problems in patient care.	3.83(1.11)
15. My boss is someone I can trust.	3.85(1.20)
20. When my colleagues are unable to decide what is right or wrong in a specific patient care situation, I find that my boss helps them.	3.66(1.05)
24. My boss is someone I respect.	4.42(0.97)
Hospital	3.54(1.10)
4. Hospital policies help me with difficult patient care issues/problems.	3.23(1.08)
8. A sense of the hospital's mission is clearly shared with nurses.	3.51(1.03)
13. The feelings and values of all parties involved in a patient care is-sue/problem are considered when planning.	3.65(1.00)
16. Conflicts are resolved openly, not avoided.	3.07(1.10)
21. There is an openness to questioning, learning and seeking creative an-swers to patient care problems.	3.59(1.09)
25. I can practice nursing in my unit in the way I believe it should be prac-ticed, ethically and legally.	4.25(0.90)
Doctors	3.23(1.16)
5. There is mutual trust between nurses and doctors.	3.32(0.94)
9. Doctors ask nurses for their opinions on treatment decisions.	2.23(1.06)
14. I participate in decisions regarding the treatment of patients under my care.	3.44(1.22)
17. Nurses and doctors here at this location respect each other's opinions, even when they disagree about what is best for patients.	3.11(1.0)
22. Nurses and doctors respect each other.	3.70(0.98)

*A: average; SD: standard deviation.*

The general ethical climate was classified as positive, among the factors, all were evaluated as positive, especially pairs with the highest average A=4.2 (0.84), except for the doctor’s factor with A=3.23 (SD=1.16), classified as negative. The item “Doctors ask nurses’ opinion on treatment decisions” presented the lowest average for the ethical climate, classified as negative (A=2.23; SD=1.06), on the other hand, the item “My boss is someone I respect” (A=4.42; SD=0.97) presented the highest average, being classified as positive. 

Table 2 presents tests comparing the means of sociodemographic/work variables and general ethical climate. 

**Table 2:** Comparison tests of means for sociodemographic/work variables and general ethical climate of emergency room health professionals. Santa Maria, RS, 2024, (n=71)

Variables	General EC
	n	A	DP	p
Sex				0.063*
Feminine	47	3.78	0.10	
Masculine	24	3.72	0.08	
Marital status				0.508*
Single	50	3.73	0.52	
With companion	21	3.85	0.75	
Have children				0.853*
Yes	46	3.75	0.55	
No	25	3.78	0.69	
Age				0.047 ***
Up to 41 years old	36	3.62	0.66	
42 years or older	35	3.9	0.49	
Training time				0.072*
Up to 16 years old	38	3.65	0.67	
17 years or older	33	3.9	0.48	
Emergency Room Sec-tor				0.515*
Adult emergency room	57	3.78	0.62	
Pediatric emergency room	14	3.68	0.52	
Professional category				0.87 **
Nurse	23	3.73	0.56	
Doctor	3	3.98	0.11	
Resident Doctors	12	3.67	0.82	
Nursing technician	33	3.8	0.57	

*T-Test used; ** Employee the test ANOVA; ***p<0.05 significant association and T-Test used for analysis. Legend: A = average; SD: standard deviation.

Female professionals (p=0.063), with a partner (p=0.508), aged over 42 years (p=0.047) and with training time over 17 years (p=0.072) presented high averages for the positive ethical climate, however, only age presented a significant association. 

Although not significant, descriptively, doctors (p=0.87) when compared to other categories, evaluated the ethical climate with higher averages, on the contrary, medical residents (p=0.87) presented lower averages for the ethical climate. 

ANOVA was performed for polytomous variables between the general ethical climate and professional category (p=0.87); education (p=0.98) and employment relationship (p=0.83), however, no association was significant. 

## Discussion

The positive perception of the general ethical climate among health professionals may be a reflection of the results of openness to questioning, ability to practice their profession and possibility of learning in search of answers to problems in patient care and organizational practices related to ethical issues employed in the unit^5^. Among the factors, the ethical climate was evaluated as negative for the physician’s factor, especially for the item that addresses their relationship with nurses, such as “Physicians ask nurses for their opinion on treatment decisions”. Other studies have identified a difficult relationship between physicians and nurses, who have difficulties in communication and dialogue when planning the diagnosis and treatment of the patient^5^,^7^. This reflects the influence of the emergency room setting itself, whose dynamics and demands fall on curative and medical work, with greater difficulty in the relationship between nursing and medicine^5^,^7^. 

An Iranian study presented a negative assessment of the physician factor, presenting a similar scenario to this study, in which the work environment is composed of an atmosphere of little cooperation and mutual relations between nurses and physicians. Among the reasons for this, one can mention the presence of social prestige and the prevalence of care still centered on the biomedical model, which is in the process of deconstruction^5^,^7^. 

Issues such as physician-centered systems in hospitals and lack of adequate staff awareness of ethical issues cause problems in the role of the nurse, which makes care more enjoyable and, as a result, creates a cooperative environment with good mutual relations among team members^5^. A Polish study indicated that cooperation with the physician is based on mutual respect, trust and active participation in treatment decisions, where managers support the nursing staff in their daily dilemmas and plan effective solutions^21^. 

The remaining factors presented a positive evaluation for the ethical climate, especially the peer factor, obtaining the highest average evaluation among health professionals. This data corresponds to the literature, indicating a good relationship between nurses and their peers, based on support, cooperation in care and resolving ethical problems together^22^. The high level of cooperation between peers can lead to a strong alliance based on professional values and responsibilities. Previous studies have reported that social support can keep colleagues focused and with the intention of collectively overcoming ethical challenges^22^,^23^. 

Furthermore, most emergency room health professionals respect their immediate superiors. This data reflects the application of tools that allow employees to make ethical decisions related to their work. It also portrays the healthy and trusting relationship between health professionals and their management. It is understood that the organization prioritizes the quality of care and employee satisfaction with the work environment^24^. Some studies have emphasized that positive perceptions of management are related to employees' psychological attachment and loyalty to their superiors, as well as to ethical leadership^22^,^24^,^25^. The exercise of ethical leadership helps the organizational climate, reducing conflicts among the health team. Leadership behavior focuses on the leader's character qualities, values, and ethical behavior, demonstrating normatively appropriate behavior through two-way communication, reinforcement, and decision-making^26^. 

A Korean study found that a positive ethical climate in the managers factor is related to the willingness of the unit management to listen and support the team in decision-making when they face ethical dilemmas in relation to a difficult situation. Through this process, health professionals come to trust and respect their managers, having a greater impact on promoting positive perceptions of the ethical climate of the unit and hospital^27^. 

By associating the ethical climate with other variables, it was possible to identify descriptively that female professionals, over 42 years of age, with a partner and with more time since graduation perceived the ethical climate with higher averages. In agreement, a Korean study interviewed 26 nurses working in the hospital area and identified that time of experience is essential to know the structure of the work and have an understanding of the services, as well as greater practice and experience regarding relationships and patient care^28^. 

More experienced professionals with more time since graduation are sensitive to culture and ethical context, since experience is related to moral competences, moral suffering and ethical evaluation, demonstrating disparity in different cultures and societies. The ethical principles that guide the health team in decision-making may vary from culture to culture, as well as between women and men, since nursing still predominates among women. However, in medicine, with the growth of interest and inclusion, there is an increase in the number of women working in the medical field^29^-^30^. 

Regarding the categories, doctors evaluated the ethical climate with high averages, when compared to the other categories. This result may indicate that the system is centered on the doctor, who feels autonomous in practicing his/her work without interference from other colleagues^5^. 

On the contrary, despite a positive evaluation, medical residents rated the ethical climate with lower averages compared to the others. This may reflect the shorter time they have worked in the unit, making it difficult to assess the work environment. On the other hand, because they work with a high workload and overload of demands, they remain in the unit for longer and, as a result, they may face more ethical issues involving care^5^. Furthermore, because they are students, they may perceive their autonomy in clinical decision-making as reduced^5^. 

This finding can be explained by ethical numbness, which is considered a loss of moral sensitivity and is believed to be one of the main ethical issues faced by some health professionals. Medical and nursing residents may become less ethically sensitive when they are very busy working in clinical settings, which can affect their perception of the ethical climate, making it more negative^6^. 

Thus, despite the positive assessment of the general ethical climate, the importance of self-reflection and greater self-awareness is understood, which can be useful in making decision-making more ethical. Health professionals engage in self-reflective behavior to be ethically competent and can be encouraged through dialogue among teammates^6^.

The study's limitations were the sample size, since data collection occurred during the health professionals' vacation period and when they were away from work due to health issues. In addition, the emergency room is predominantly staffed by nurses and nursing technicians, so the main result is not sufficiently counterbalanced by the doctors' opinion, since there are few of them in the entire sample. 

## Conclusions

Emergency room health professionals assessed the general ethical climate and its factors as positive, however, the physician factor was perceived as negative. Descriptively, female health professionals, over 42 years of age, with a longer training period and with a partner perceived the ethical climate as positive, however only age showed a significant association. 

Research and clinical practice should explore supportive interventions and improve the ethical environment at the system level. Understanding the perceptions of health professionals can provide crucial information for managers to optimize medical policies and procedures, as well as undertake ethics education and institutional support. In addition, the use of ECP was essential for diagnosing the ethical climate in the emergency room, being an effective tool to assist in data collection, allowing it to be remote and accessed from any location. 

To improve the ethical climate among physicians, small meetings or conferences should be held periodically to exchange opinions and experiences with physicians and nurses on patient care and ethical issues. In addition, different actions are proposed related to other domains, for example, workshops, seminars or periodic counseling to develop leadership skills among nurses. 
